# Health service utilization following symptomatic respiratory tract infections and influencing factors among urban and rural residents in Anhui, China

**DOI:** 10.1017/S1463423619000896

**Published:** 2019-12-10

**Authors:** Shiyu Xu, Xuemeng Dong, Rongyao Zhou, Xingrong Shen, Rui Feng, Jing Cheng, Jing Chai, Paul Kadetz, Debin Wang

**Affiliations:** 1 School of Health Service Management, Anhui Medical University, Hefei, China; 2 The First Affiliated Hospital of Anhui Medical University, Hefei, China; 3 University Library, Anhui Medical University, Hefei, China; 4 Department of Anthropology, Drew University, Madison, NJ, USA

**Keywords:** China, healthcare utilization, influencing factors, respiratory tract infections

## Abstract

**Aim:**

This study seeks to identify healthcare utilization patterns following symptomatic respiratory tract infections (RTIs) and the variables that may influence these patterns.

**Background:**

RTIs are responsible for the bulk of the primary healthcare burden worldwide. Yet, the use of health services for RTIs displays great discrepancies between populations. This research examines the influence of social demographics, economic factors, and accessibility on healthcare utilization following RTIs.

**Methods:**

Structured interviews were administered by trained physicians at the households of informants selected by cluster randomization. Descriptive and multivariate binary logistic regression analysis was performed to assess healthcare utilization and associated independent variables.

**Findings:**

A total of 60 678 informants completed the interviews. Of the 2.9% informants exhibiting upper RTIs, 69.5–73.9% sought clinical care. Healthcare utilization rates for common cold, influenza, nine acute upper RTIs, and overall RTIs demonstrate statistically significant associations with the variables of age, type of residence, employment, medical insurance, annual food expenditure, distance to medical facilities, and others. The odds ratios for healthcare utilization rates varied substantially, ranging from 0.026 to 9.364. More than 69% of informants with RTIs sought clinical interventions. These findings signify a marked issue with the large amount of healthcare for self-limited RTIs.

## Introduction

Respiratory tract infections (RTIs) place a substantial burden on healthcare resources and society (Thompson *et al*., 2013; Lenoir-Wijnkoop *et al*., 2019). Globally, over three million people die annually from RTIs, and RTIs have become a major factor contributing to the death of children under age 5 (GBD 2013 Mortality and Causes of Death Collaborators, 2015; Word Health Organization, 2017). RTIs have become a persistent, pervasive, global public health issue that results in substantial burdens on patients, their families, and society at large. The most common RTIs are upper RTIs (URTIs), which include the common cold, influenza, acute pharyngitis, sore throat, acute tonsillitis, acute suppurative tonsillitis, acute laryngitis, acute bronchitis, acute laryngopharyngitis, and acute URTI (AURTI). Leder *et al*. (2003) estimated an average of 2.2 episodes of URTIs per person per year in Melbourne, Australia, with a mean duration of 6.3 days. This frequency is equivalent to experiencing respiratory symptoms every seven weeks for a period of two days (Leder *et al*., 2003). However, symptoms of URTIs are relatively limited, consisting mainly of sore throat, fever, dry or productive cough, rhinorrhea with or without pus, shortness of breath, headache, general discomfort, and earache or tinnitus (Fischer *et al*., 2005; Lu *et al*., 2013; Diao *et al*., 2018). RTIs are the main reason for primary healthcare visits. But only a few cases of RTIs are caused by bacterial infection (Harnden *et al*., 2007). Research and guidelines worldwide suggest that the use of antibiotics is not needed for non-specific respiratory infections (Meropol *et al*., 2009).

Patients with RTIs differ widely in their healthcare-seeking behaviors. Some RTI patients seek no interventions and wait for self-remission. Other patients manage their symptoms with non-medical practices, such as remaining indoors, staying in bed, and drinking hot beverages (Halvorsen *et al*., 2014). Still other patients choose self-medication (eg, by taking leftover prescription medicines and/or buying medicines and antibiotics from retail pharmacies without a prescription) (Word Health Organization, 2018), or seeking professional intervention. Data about healthcare utilization for RTIs in China are scarce, though limited research reported marked discrepancies between China and other countries and between different population groups within China. In the United States, only 55% patients with cough and common cold eventually sought professional care, but in another study by us, 78.8% of patients with RTIs sought treatment from clinics or bought medicine from medical shops (Blaiss *et al*., 2015; Diao *et al*., 2018). Compared with the UK and other European countries, RTI patients in China reported more frequent symptoms and higher and earlier help-seeking from a doctor following the infection (Chai *et al*., 2019). Most importantly, there are reports that antibiotic use is very high for RTI patients in various clinical settings in China (Dong *et al*., 2008; Yin, 2009; Zhao *et al*., 2013), and excessive use of antibiotics is closely linked to antibiotic resistance (Turnidge and Christiansen, 2005; Costelloe *et al*., 2010). A systematic review in 2014 documented as much as 83.7% of antibiotic use among RTI outpatients in China (Li *et al*., 2016). This rate of antibiotic use is significantly higher than recommended by the European Surveillance of Antimicrobial Consumption (Adriaenssens *et al*., 2011). Studies exploring reasons for patients’ varied health service use, as well as for the prevalence of antibiotic prescriptions in China, are also lacking. According to our systemic review of the literature, few publications focus primarily on the factors impacting healthcare and antibiotic use following URTIs/RTIs, though a number of papers that assessed the determinants of service utilization for all diseases also included RTIs (Vazquez-Lago *et al*., 2012; May *et al*., 2014). The current research aims to bridge these gaps in the literature.

## Methods

### Study design

This cross-sectional quantitative research comprised four main groups of variables: (a) social demographics that include age, sex, marital status, education, employment, and registered place of residence (or the *Hukou*, which acts as a kind of ‘internal passport’ that can restricts welfare benefits to one’s place of birth); (b) economic factors, including annual income, household expenditure, food expenditure, and medical insurance; (c) physical and financial accessibility of healthcare services, including distance and time needed for transport to medical facilities, perceived changes in accessibility, and cost of healthcare services, and perceived doctor–patient relationships; and (d) healthcare utilization following RTIs, including the common cold, influenza, acute pharyngitis, sore throat, acute tonsillitis, acute suppurative tonsillitis, acute laryngitis, acute bronchitis, acute laryngopharyngitis, AURTI, and viral URTI.

### Selection of site and subjects

This study extracted data from an extensive Household Health Service Survey conducted in 2014 in Anhui Province, China. A stratified-clustered randomized sampling for both sites and participating residents proceeded in two steps. In step 1, all the counties and cities in Anhui province were grouped into southern, northern, and middle areas. In step 2, sites were randomly selected according to random numbers assigned to the sites using a Microsoft Excel worksheet. The sites selected included: (a) six counties and six cities from each of the north, middle, and south areas of Anhui (amounting to 18 counties and 18 cities in total); (b) five communities from each of the counties or cities; and (c) 120 households from each of the communities. Sample inclusion criteria included the requirement that all members within the selected households needed to have maintained residence in their local community for over six months of the year of the study. Informants also needed to be able to answer all survey questions to be eligible to participation in this study.

### Data collection

Data were collected via face-to-face interviews from April to November 2014. Structured interviews were administered by trained village- or township-level physicians at respondents’ households. The head of the house, or the household member who knew most members within the household, served as the informant for each household.

Data about diagnosis or classification of illness or symptoms in the two weeks prior to the interview were based on the judgment of the researchers who referred to an abridged checklist of common diseases (including respiratory infections) that provided disease names, ICD-10 codes, and key indications for making diagnoses/classifications. Measures taken to ensure data quality included (a) training and examination of field data collectors; (b) daily checks by quality supervisors of all questionnaires completed during the day; (c) re-test of 5% of randomly selected sample of subjects; and (d) feedback of errors found via daily checks and re-tests.

### Data analysis

Data were double-entered using EPIDATA v.3.1 and analyzed with SPSS v.10.01. Data analysis included descriptive analysis and multivariate binary logistic regression modeling. Descriptive analysis calculated service utilization rates for common cold, the nine AURTIs (acute pharyngitis, sore throat, acute tonsillitis, acute suppurative tonsillitis, acute laryngitis, acute bronchitis, acute laryngopharyngitis, AURTI, and viral URTI), influenza, other RTI symptoms and overall RTI symptoms by different subgroups (eg, age groups, gender groups). The power of differences between these groups was estimated using a two-sided chi-square test of the null hypothesis. Multivariate binary logistic regression adopted the ‘enter’ approach and used the utilization rates for overall and specific categories of RTIs as the dependent variable, and demographic, social, economic, and accessibility of health service as independent variables. Here healthcare utilization rate due to a specific RTI (say common cold) stands for the ratio of patients who had sought help for the RTI from doctors to the total patients who had reported the same RTI.

## Results

A total of 60 678 respondents completed the structured interview, of which 29 980 were males and 30 698 females; with 30 597 urban dwellers and 30 081 rural dwellers. Of the total respondents, 2.9% reported symptoms of RTIs (9.1% for children under five) in the final two weeks of the study. As illustrated in Table 1, 69.2% of the informants sought professional care, with an average of 1.5 visits to a clinic per patient per infection. Patients with a diagnosis from the nine AURTIs demonstrated the highest health service utilization (73.9%), followed by those with influenza (72.0%) and the common cold (69.5%) and other RTIs (including pneumonia and emphysema comprising 57.9%). These healthcare utilization rates demonstrate statistically significant differences between all age subgroups (*P* < 0.05), as well as between other demographic categories including place of residency, urban versus rural residency, and education and employment subgroups (*P* < 0.05). However, the variations between sex and marriage groups were not statistically significant. Age demonstrated a consistent L-shaped trend for all RTIs, in which utilization rates were highest for the under-five group, dropped rapidly for the 25–34-year age group, and increased gradually for older age groups. Education also demonstrated a consistent trend for almost all RTIs, with more educated informants being less likely to use healthcare services. With regard to other factors studied, (a) healthcare utilization rates were higher among rural compared to urban residents; (b) higher in North Anhui compared to other areas of Anhui; and (c) higher among employed versus retired or unemployed.

**Table 1.** Use of healthcare services following RTIs as per demographic factors

	Common cold	Nine AURTIs	Influenza	Others	Total
Age group					
<5	163 (81.1%)	111 (87.4%)	55 (84.6%)	19 (90.5%)	348 (84.1%)
5–14	77 (65.8%)	66 (80.5%)	27 (77.1%)	3 (100.0%)	173 (73.0%)
15–24	31 (62.0%)	20 (80.0%)	8 (61.5%)	6 (85.7%)	65 (68.4%)
25–34	41 (59.4%)	24 (66.7%)	8 (44.4%)	3 (75.0%)	76 (59.8%)
35–44	64 (61.0%)	42 (68.9%)	17 (65.4%)	16 (57.1%)	139 (63.2%)
45–54	96 (67.6%)	56 (63.6%)	27 (55.1%)	16 (50.0%)	195 (62.7%)
55–64	85 (65.4%)	48 (67.6%)	33 (71.7%)	50 (61.0%)	216 (65.7%)
65–74	57 (74.0%)	38 (64.4%)	23 (88.5%)	46 (51.1%)	164 (65.1%)
>75	27 (87.1%)	34 (75.6%)	13 (86.7%)	38 (52.1%)	112 (68.3%)
*P*	0.000	0.001	0.002	0.010	0.000
Gender					
Male	316 (72.3%)	198 (72.3%)	112 (74.2%)	98 (54.7%)	724 (69.5%)
Female	325 (67.0%)	241 (75.3%)	99 (69.7%)	99 (61.5%)	764 (69.0%)
*P*	0.072	0.399	0.450	0.184	0.785
Registered residence type					
Urban	326 (63.8%)	196 (67.8%)	72 (63.7%)	88 (59.1%)	682 (64.2%)
Rural	315 (76.6%)	243 (79.7%)	139 (77.2%)	109 (57.1%)	806 (74.1%)
*P*	0.000	0.001	0.029	0.754	0.000
Areas					
North of Anhui	338 (71.8%)	100 (87.7%)	86 (78.2%)	83 (68.0%)	607 (74.3%)
Middle of Anhui	125 (69.4%)	117 (67.2%)	79 (72.5%)	51 (45.5%)	372 (64.7%)
South of Anhui	178 (65.7%)	222 (72.5%)	46 (62.2%)	63 (59.4%)	509 (67.2%)
*P*	0.222	0.000	0.042	0.002	0.000
Marriage					
Never married	25 (64.1%)	27 (87.1%)	5 (55.6%)	4 (44.4%)	61 (69.3%)
Married	559 (69.4%)	368 (72.7%)	180 (71.7%)	153 (59.8%)	1260 (69.3%)
Other	56 (72.7%)	43 (76.8%)	26 (81.2%)	39 (52.7%)	164 (68.6%)
*P*	0.633	0.183	0.538	0.442	0.941
Education					
0 year	319 (73.2%)	242 (80.7%)	120 (80.0%)	95 (58.3%)	776 (74.0%)
1–5 years	111 (68.1%)	84 (73.0%)	42 (70.0%)	61 (61.6%)	298 (68.2%)
6–9 years	133 (66.5%)	75 (71.4%)	26 (61.9%)	28 (50.0%)	262 (65.0%)
>9 years	77 (63.1%)	37 (50.7%)	23 (57.5%)	12 (57.1%)	149 (58.2%)
*P*	0.095	0.000	0.030	0.530	0.000
Employment					
Employed	495 (69.4%)	355 (76.8%)	171 (71.2%)	127 (63.2%)	114 8 (71.0%)
Retired	25 (58.1%)	13 (40.6%)	3 (100.0%)	15 (51.7%)	56 (52.3%)
Unemployed or never employed	114 (73.1%)	62 (68.9%)	37 (77.1%)	52 (48.6%)	265 (66.1%)
*P*	0.168	0.000	0.358	0.033	0.000
Total	641 (69.5%)	439 (73.9%)	211 (72.0%)	197 (57.9%)	1488 (69.2%)

Note: Power of differences between groups was estimated using the chi-square test.

Table 2 illustrates healthcare utilization rates for informants with RTIs according to economic status, including medical insurance, annual income, annual household expenditure, and annual food expenditure. Variations in healthcare utilization rates between most of these subgroups were tested with no demonstration of statistical significance, except for medical insurance and annual food expenditure categories. For food expenditure, utilization rates displayed inverse relationships with healthcare utilization. For example, the overall healthcare utilization for RTIs ranged from 62.3% (for those with the highest food expenditure) to 72.8% (for those with the lowest food expenditure). Healthcare utilization for the common cold ranged from 59.5% to 75.2%; and for the nine AURTIs from 64.7% to 77.5%. Healthcare utilization was also correlated with the patient’s type of medical insurance. For example, members of the Urban–Rural Joint Medical Care System (whose rates for reimbursement is the same for both urban and rural members) witnessed the highest healthcare utilization rates (ie, 85.4% for common cold, 81.4% for the nine AURTIs, and 79.4% for overall RTIs), while members of the city employee medical insurance (which is only applicable to urban employees) displayed the lowest healthcare utilization rates (ie, 48.0% for common cold, 38.8% for the nine AURTIs, and 45.0% for overall RTIs).

**Table 2.** Health service utilization rates following RTIs as per economic factors

	Common cold	Nine AURTIs	Influenza	Others	Total
Medical insurance					
City employee medical insurance	36 (48.0%)	19 (38.8%)	9 (50.0%)	13 (44.8%)	77 (45.0%)
City resident’s medical insurance	55 (64.7%)	35 (53.8%)	9 (64.3%)	14 (56.0%)	113 (59.8%)
New rural medical care system	412 (74.0%)	293 (80.9%)	158 (74.2%)	136 (58.6%)	999 (73.2%)
Urban–rural joint medical care system	41 (85.4%)	35 (81.4%)	11 (73.3%)	21 (70.0%)	108 (79.4%)
Other medical insurance	20 (71.4%)	6 (85.7%)	8 (80.0%)	2 (100.0%)	36 (76.6%)
≥2 medical insurances	77 (59.7%)	51 (75.0%)	16 (69.6%)	11 (50.0%)	155 (64.0%)
*P*	0.000	0.000	0.193	0.308	0.000
Annual income					
<19 000 RMB	149 (72.7%)	92 (71.9%)	43 (87.8%)	76 (55.1%)	360 (69.2%)
19 000–30 000 RMB	201 (73.6%)	121 (76.6%)	59 (70.2%)	51 (56.0%)	432 (71.3%)
30 001–50 000 RMB	167 (65.2%)	127 (77.0%)	63 (69.2%)	40 (59.7%)	397 (68.6%)
>50 000 RMB	124 (66.3%)	99 (69.2%)	46 (69.7%)	29 (70.7%)	298 (68.2%)
*P*	0.099	0.350	0.082	0.329	0.679
Annual household expenditure					
≤15 400 RMB	160 (72.1%)	107 (79.3%)	57 (79.2%)	86 (54.8%)	410 (70.0%)
15 401–24 400 RMB	156 (71.6%)	96 (73.8%)	44 (71.0%)	38 (58.5%)	334 (70.3%)
24 401–36 600 RMB	163 (71.5%)	110 (75.3%)	69 (75.0%)	36 (59.0%)	378 (71.7%)
>36 600 RMB	162 (64.0%)	126 (68.9%)	41 (64.1%)	37 (67.3%)	366 (65.9%)
*P*	0.163	0.205	0.234	0.451	0.192
Annual food expenditure					
<5000	179 (75.2%)	138 (77.5%)	62 (79.5%)	92 (56.8%)	471 (71.8%)
5000–10 000	229 (71.8%)	144 (79.1%)	64 (69.6%)	69 (65.1%)	506 (72.4%)
10 000–15 000	111 (69.4%)	66 (70.2%)	38 (69.1%)	11 (39.3%)	226 (67.1%)
≥15 000	122 (59.5%)	90 (64.7%)	47 (69.1%)	24 (57.1%)	283 (62.3%)
*P*	0.002	0.015	0.469	0.082	0.001
Total	641 (69.5%)	439 (73.9%)	211 (72.0%)	197 (57.9%)	1488 (69.2%)

Note: Power of differences between groups was estimated using the chi-square test.

Table 3 presents health service utilization rates following URTIs and total RTIs according to accessibility to healthcare services. Physical accessibility includes the distance and time to reach medical facilities. Whereas, financial accessibility includes perceived changes in costs of health services. In general, statistically significant differences were demonstrated in healthcare utilization rates between almost all subgroups, except for the ‘time to reach medical facilities’ group. Distance to medical facilities displayed positive correlations with healthcare utilization for the common cold (ranging from 66.4% for shortest distance to medical facilities, to 77.8% for longest distance to medical facilities), the nine AURTIs (from 67.7% to 84.2%, respectively), influenza (from 63.6% to 88.2%), and overall RTIs (from 64.6% to 77.2%, respectively). A substantial and consistently increasing correlation was identified between the informants’ perceived improvement in healthcare access and their perceived decreases in healthcare costs for almost all the URTI and total RTI groupings. Finally, those informants who perceived the doctor–patient relationship as most similar to that of parents and children were most likely to exhibit higher healthcare utilization rates.

**Table 3.** Health service utilization rates following RTIs by accessibility of health service utilization

	Common cold	Nine AURTIs	Influenza	Others	Total
Distance to medical facilities					
<1 km	332 (66.4%)	210 (67.7%)	96 (63.6%)	80 (53.3%)	718 (64.6%)
1–2 km	147 (71.7%)	107 (78.7%)	52 (75.4%)	54 (58.7%)	360 (71.7%)
2–3 km	119 (77.8%)	85 (84.2%)	48 (85.7%)	40 (58.8%)	292 (77.2%)
≥3 km	38 (65.5%)	36 (81.8%)	15 (88.2%)	22 (78.6%)	111 (75.5%)
*P*	0.033	0.002	0.009	0.098	0.000
Travel time to medical facilities					
<5 min	260 (69.9%)	185 (71.7%)	77 (67.5%)	58 (53.7%)	580 (68.1%)
5–10 min	192 (67.1%)	147 (76.6%)	78 (78.0%)	69 (58.5%)	486 (69.8%)
10–15 min	77 (71.3%)	34 (72.3%)	27 (77.1%)	21 (63.6%)	159 (71.3%)
≥15 min	107 (71.3%)	71 (76.3%)	29 (69.0%)	48 (60.8%)	255 (70.1%)
*P*	0.792	0.638	0.357	0.673	0.752
Perceived changes in the accessibility of health services					
Improved greatly	319 (71.8%)	211 (79.6%)	99 (74.4%)	88 (56.4%)	717 (71.8%)
Improved slightly	250 (70.2%)	165 (70.8%)	74 (70.5%)	90 (62.5%)	579 (69.1%)
Other	68 (58.1%)	62 (66.0%)	38 (71.7%)	18 (47.4%)	186 (61.6%)
*P*	0.015	0.013	0.792	0.217	0.004
Perceived changes in the cost of health services					
Improved greatly	197 (76.4%)	151 (79.5%)	62 (79.5%)	57 (54.3%)	467 (74.0%)
Improved Slightly	251 (70.5%)	156 (78.0%)	90 (71.4%)	83 (58.5%)	580 (70.4%)
Other	189 (62.4%)	131 (64.9%)	59 (67.8%)	56 (61.5%)	435 (63.7%)
*P*	0.001	0.001	0.267	0.576	0.000
Perceived doctor–patient relationships					
Parent–child relationship	33 (80.5%)	28 (80.0%)	7 (87.5%)	10 (66.7%)	78 (78.8%)
Friend	401 (71.7%)	289 (77.3%)	139 (74.3%)	123 (58.3%)	952 (71.5%)
Workmates	30 (69.8%)	16 (61.5%)	12 (85.7%)	7 (46.7%)	65 (66.3%)
Buyer–seller relationship	81 (56.2%)	49 (60.5%)	30 (60.0%)	23 (47.9%)	183 (56.7%)
Other	93 (71.0%)	57 (73.1%)	23 (71.9%)	33 (67.3%)	206 (71.0%)
*P*	0.003	0.014	0.169	0.236	0.000
Total	641 (69.5%)	439 (73.9%)	211 (72.0%)	197 (57.9%)	1488 (69.2%)

Note: Power of differences between groups was estimated using the chi-square test.

Table 4 presents summary statistics of multivariate binary logistic regression analysis using healthcare utilization rates due to common cold, influenza, the nine AURTIs, other symptoms of RTIs, and the overall RTIs as the dependent variable, and 11 independent variables, including demographic factors, economic characteristics, and accessibility of health service utilization. All the utilization rates demonstrated statistically significant associations with 5–8 of the 11 independent variables. The category of age displayed significant links to all of the main categories of URTI and total RTI groupings. However, the categories of education, employment, perceived changes in accessibility of health services, and perceived changes in the cost of health services displayed no correlation with any RTI grouping. However, the remaining variables (ie, type and place of residence, type of medical insurance, food expenditures, physical accessibility to healthcare, and doctor–patient relationship) illustrated a correlation with only some of the RTIs. The consistent trends in utilization rates among subgroups of age, and distance traveled to medical facilities, presented in Tables 1 and 3 were also observed in the multivariate regression model. The odds ratios for healthcare utilization rates ranged from 0.026 (for other RTIs among the 35–44-year group compared with the under-five group) to 9.364 (for the nine AURTIs among the respondents with a distance to the closest caregiver of ≥3 km compared with those <1 km from professional service).

**Table 4.** Logistic regression modeling of relationships between healthcare utilization following RTIs and common influential factors

	Common cold		Nine AURTIs		Influenza		Others		Total	
	OR (95% CI)	*P*	OR (95% CI)	*P*	OR (95% CI)	*P*	OR (95% CI)	*P*	OR (95% CI)	*P*
Age group (<5 serves as reference)										
5–14	0.46 (0.26, 0.81)	0.006	0.59 (0.25, 1.35)	0.251	0.57 (0.15, 2.02)	0.392	1.45E7 (0.00)	0.999	0.54 (0.36, 0.82)	0.003
15–24	0.30 (0.09, 0.59)	0.002	0.32 (0.06, 1.81)	0.824	1.28 (0.06, 26.25)	0.431	4.83E7 (0.00)	0.999	0.34 (0.17, 0.69)	0.008
25–34	0.29 (0.13, 0.64)	0.002	0.28 (0.08, 0.96)	0.042	0.26 (0.05, 1.47)	0.173	0.06 (0.01, 1.92)	0.401	0.27 (0.16, 0.46)	0.001
35–44	0.37 (0.18, 0.73)	0.005	0.23 (0.08, 0.68)	0.034	0.32 (0.07, 1.49)	0.305	0.03 (0.01, 0.29)	0.012	0.31 (0.19, 0.49)	0.001
45–54	0.44 (0.23, 0.82)	0.009	0.17 (0.07, 0.45)	0.001	0.19 (0.05, 0.70)	0.038	0.03 (0.01, 0.24)	0.004	0.28 (0.19, 0.43)	0.001
55–64	0.38 (0.21, 0.68)	0.001	0.24 (0.10, 0.58)	0.001	0.34 (0.10, 1.13)	0.207	0.05 (0.01, 0.38)	0.005	0.32 (0.22, 0.47)	0.001
65–74	0.61 (0.29, 1.29)	0.210	0.33 (0.12, 0.88)	0.013	0.82 (0.15, 4.59)	0.725	0.03 (0.01, 0.26)	0.001	0.33 (0.21, 0.51)	0.001
>74	1.32 (0.38, 4.57)	0.681	0.54 (0.18, 1.64)	0.167	1.35 (0.12, 15.26)	0.823	0.03 (0.01, 0.27)	0.001	0.40 (0.24, 0.67)	0.001
Residency type (urban serves as reference)										
Rural	1.69 (1.18, 2.43)	0.005	1.13 (0.67, 1.91)	0.790	2.39 (1.08, 5.29)	0.046	0.97 (0.52, 1.83)	0.904	1.43 (1.14, 1.79)	0.003
Areas (middle of Anhui serves as reference)										
North of Anhui	1.18 (0.76, 1.84)	0.496	2.03 (0.92, 4.49)	0.073	1.55 (0.65, 3.67)	0.293	4.09 (1.94, 8.64)	0.004	1.55 (1.19, 2.01)	0.002
South of Anhui	0.68 (0.43, 1.09)	0.089	0.81 (0.48, 1.38)	0.333	0.36 (0.14, 0.91)	0.022	2.22 (1.09, 4.51)	0.047	0.89 (0.69, 1.15)	0.459
Education (0 year serves as reference)										
1–5 years	0.99 (0.59, 1.68)	0.983	1.56 (0.76, 3.21)	0.295	0.41 (0.14, 1.22)	0.108	1.46 (0.76, 2.82)	0.207	1.20 (0.89, 1.62)	0.285
6–9 years	1.41 (0.80, 2.47)	0.250	1.55 (0.67, 3.58)	0.621	0.80 (0.24, 2.74)	0.561	0.85 (0.35, 2.10)	0.521	1.28 (0.90, 1.81)	0.230
>9 years	1.59 (0.80, 3.13)	0.165	0.63 (0.25, 1.63)	0.139	0.53 (0.13, 2.26)	0.511	0.60 (0.15, 2.37)	0.317	1.08 (0.70, 1.64)	0.771
Employment (employed serves as reference)										
Retired	1.01 (0.44, 2.30)	0.979	0.34 (0.10, 1.12)	0.133	1.14E9 (0.00)	0.999	1.89 (0.52, 6.86)	0.361	0.88 (0.52, 1.50)	0.698
Unemployed or never employed	1.05 (0.65, 1.68)	0.752	1.04 (0.52, 2.06)	0.993	2.32 (0.69, 7.85)	0.199	0.66 (0.34, 1.28)	0.247	0.95 (0.72, 1.27)	0.697
Medical insurance (city employee medical insurance serves as reference)										
City resident’s medical insurance	2.29 (1.02, 5.14)	0.045	0.73 (0.27, 1.97)	0.549	0.49 (0.06, 4.23)	0.704	1.31 (0.28, 6.22)	0.762	1.31 (0.79, 2.17)	0.336
New rural medical care system	2.33 (1.08, 5.06)	0.032	2.02 (0.75, 5.42)	0.156	0.65 (0.11, 3.84)	0.747	1.28 (0.30, 5.39)	0.748	1.70 (1.05, 2.77)	0.026
Urban–rural joint medical care system	4.40 (1.43, 13.51)	0.007	1.02 (0.26, 4.00)	0.882	1.05 (0.11, 9.85)	0.902	2.08 (0.35, 12.28)	0.760	1.98 (1.05, 3.76)	0.031
Other medical insurance	2.08 (0.67, 6.43)	0.198	1.77 (0.15, 21.37)	0.792	5.35E8 (0.00)	0.999	4.00E7 (0.00)	0.999	1.96 (0.81, 4.75)	0.152
≥2 medical Insurances	1.22 (0.55, 2.73)	0.545	1.23 (0.41, 3.65)	0.580	0.60 (0.07, 4.90)	0.841	0.76 (0.14, 4.21)	0.700	0.95 (0.57, 1.60)	0.988
Annual food expenditure (<500 RMB as reference)										
5000–10 000 RMB	0.90 (0.56, 1.45)	0.808	1.12 (0.57, 2.20)	0.487	0.37 (0.14, 1.01)	0.145	0.99 (0.47, 2.11)	0.259	0.94 (0.70, 1.26)	0.679
10 000–15 000 RMB	0.89 (0.48, 1.66)	0.738	0.87 (0.38, 2.00)	0.768	0.62 (0.20, 1.97)	0.567	0.12 (0.03, 0.44)	0.032	0.75 (0.51, 1.10)	0.517
≥15 000 RMB	0.48 (0.26, 0.91)	0.023	0.74 (0.30, 1.81)	0.891	0.26 (0.08, 1.01)	0.079	0.26 (0.08, 0.88)	0.041	0.61 (0.40, 0.91)	0.037
Distance to medical facilities (<1 km serves as reference)										
1–2 km	1.20 (0.78, 1.85)	0.376	1.31 (0.72, 2.37)	0.577	1.27 (0.49, 3.29)	0.746	1.08 (0.53, 2.20)	0.447	1.19 (0.91, 1.56)	0.379
2–3 km	1.95 (1.14, 3.36)	0.014	2.43 (1.15, 5.14)	0.039	3.43 (1.11, 10.67)	0.033	0.99 (0.42, 2.33)	0.513	1.90 (1.37, 2.64)	0.001
≥3 km	1.43 (0.71, 2.87)	0.301	3.05 (1.09, 8.53)	0.077	9.26 (1.21, 70.81)	0.035	2.38 (1.20, 11.35)	0.022	2.12 (1.34, 3.35)	0.008
Perceived changes in healthcare access (‘improved greatly’ serves as reference point)										
Improved slightly	1.24 (0.84, 1.82)	0.240	0.76 (0.41, 1.40)	0.351	1.04 (0.42, 2.56)	0.871	1.94 (0.93, 4.06)	0.356	1.09 (0.85, 1.41)	0.632
Other	0.92 (0.54, 1.56)	0.817	0.73 (0.36, 1.50)	0.338	2.07 (0.60, 7.13)	0.175	0.86 (0.32, 2.29)	0.450	0.92 (0.66, 1.28)	0.543
Perceived changes in the cost of the health services (‘improved greatly’ serves as reference point)										
Improved slightly	0.75 (0.48, 1.18)	0.209	1.23 (0.62, 2.45)	0.592	0.92 (0.33, 2.56)	0.684	0.69 (0.32, 1.51)	0.441	0.89 (0.66, 1.18)	0.396
Other	0.66 (0.41, 1.08)	0.088	0.94 (0.46, 1.91)	0.800	0.82 (0.24, 2.83)	0.547	1.22 (0.49, 3.03)	0.438	0.84 (0.61, 1.15)	0.295
Perceived doctor–patient relationships (parent–child relation serves as reference)										
Friend	0.72 (0.30, 1.74)	0.501	0.57 (0.19, 1.68)	0.354	0.10 (0.01, 1.35)	0.086	0.62 (0.17, 2.32)	0.803	0.63 (0.37, 1.08)	0.096
Workmates	0.99 (0.31, 3.18)	0.987	0.48 (0.11, 2.04)	0.317	0.09 (0.01, 2.41)	0.137	0.16 (0.02, 1.03)	0.121	0.67 (0.33, 1.35)	0.236
Buyer–seller relationship	0.43 (0.17, 1.14)	0.097	0.40 (0.12, 1.30)	0.175	0.05 (0.01, 0.78)	0.036	0.27 (0.06, 1.19)	0.165	0.41 (0.23, 0.73)	0.002
Other	0.75 (0.28, 1.98)	0.598	0.47 (0.14, 1.57)	0.354	0.19 (0.01, 3.39)	0.197	0.78 (0.18, 3.37)	0.812	0.68 (0.37, 1.22)	0.186

Note: Multivariate binary logistic regression adopted the ‘enter’ approach.

## Discussion

### Main findings of this study

This research uncovers useful data to better understand RTI-related healthcare use among urban and rural residents in Anhui, China. The study found that, on average, 2.9% of all residents examined experienced symptoms of RTIs in the past two weeks, and 69.2% of these symptomatic patients sought care from a doctor. These estimations suggest that RTIs incur enormous health service burdens.

This study identified an L-shaped link between service utilization rate and age (in which utilization was the highest from under-five years, followed by a rapid decrease and then a gradual increase from around the age of 35). This typical pattern of age-related trends for all RTIs combined may be explained mainly by immunity changes. Compared with the common cold and the nine AURTIs, influenza did not demonstrate a clear L-shaped trend. This may be explained by the fact that influenza develops when the pathogen (a virus) has mutated to escape the existing immunity of a large proportion of people. So the chances of developing influenza are equal across age groups. The relatively lower service use due to influenza for the 45–54 age group may be an outcome of the demands on senior-level employment positions filled by this age group who thereby may be the age group least capable of re-arranging their work schedule and seeking healthcare.

The association between service use and distance to health facilities also presents interesting trends. This association was less significant for those who lived <1 km from the health facility and peaked for those who lived at a distance of 2–4 km away. One possible explanation for these trends is that people living in community centers are at the highest risk of getting RTIs, due to the high population density and more frequent person-to-person contacts. Health facilities are generally located near community centers, being about 2–4 km from rather than right at these centers. In other words, people who live around health facilities are generally apart from crowded community centers.

The findings concerning the correlations between health service utilization following RTIs, or specific categories of URTIs and other variables studied (including type and place of residence, type of medical insurance, income, food expenditures, and perceived doctor–patient relationship), also merit attention. These relationships may again reflect the complex interactions between host immunity, pathogen exposure, resource availability, and infection recognition. In addition, the large discrepancies in the odds ratios of healthcare utilization due to different categories of RTIs, among different subgroups (ranging from 0.026 to 9.364), may suggest a need for tailored thinking or strategies in addressing related issues.

### What is already known on this topic

The finding that RTIs are very prevalent and a large proportion of infection episodes leads to health service use is consistent with research across various countries (Yawson *et al*., 2012; Gounder *et al*., 2017; Dowell *et al*., 2017). However, the implications of these findings for clinical practice and policy-making have yet to be fully explored. All developed and a majority of developing countries have issued clinical guidelines concerning the diagnosis and treatment of diabetes, hypertension, cancer, and other non-communicable diseases. Similar guidelines concerning the management of RTIs are hardly retrievable from the literature. Although the US Centers for Disease Control have developed guidelines for antibiotics use for adult acute respiratory infections (Harris *et al*., 2016), and the China Ministry of Health developed guidelines for influenza management (Zhong *et al*., 2011), they were driven by other related issues (eg, antibiotics use and influenza report and monitoring) rather than RTIs themselves. The ‘self-limiting’ feature seems to have masked the huge service burden and even the excessive mortality from RTIs, leading to a widespread underestimation of the importance of these infections. There seems to be an important flaw with our contemporary strategy toward RTIs. The self-limiting feature combined with the difficulty in finding cures seem to have not only prevented us from reaching well-recognized guidelines for treating RTIs but also deterred us from identifying effective strategies for reducing health service burden and other indirect harms due to the infections.

The L-shaped link between service utilization rate and age (in which utilization was greatest for the under-five group, followed by a rapid decrease and then a gradual increase at around the age of 35) is also consistent, to some degree, with previous studies (Keene and Li, 2005; Geitona *et al*., 2007; Nouraei Motlagh *et al*., 2015). A number of studies reported that young pediatric patients are susceptible to most kinds of pathogens and are at the highest risk of contracting RTIs (Liao *et al*., 2015). Causes underlining these L-shaped relations have not been fully addressed. As an infant develops immunity against common RTIs, the opportunity for infection transmission decreases rapidly. Immunity continues to develop, tapering off at a plateau during teenage years. Starting from late adulthood, immunity begins to decrease gradually (Pawelec, 2006).

Previous studies have also revealed similar associations between service use and distance to health facilities. For instance, NoorAli R and colleagues reported that residents living at a distance of <4 km from a government facility made 22% less use of that facility than those living ≥4 km away (NoorAli *et al*., 1999). Müller *et al.* (1998) found that attendance decreased markedly with distance, for example, a 50% decrease at 3.5 km.

### What this study adds to the field

To our knowledge, data about healthcare use for RTIs among Chinese respondents are scarce, even though China has the largest population in the world. This study helps to bridge this information gap. This research reveals that 2.9% of all residents examined experienced symptoms of RTIs in the past two weeks, and >69% of informants with URTIs sought clinical treatments. The large amount of healthcare use for self-limited acute RTIs from this study and the high antibiotics prescription rates for attendees with RTIs from other studies in China point to a clear need for strategies to contain the service burden and other related adverse effects. Such strategies may include (a) promoting health literacy with respect to the burden of RTIs; (b) encouraging vacuum treatment or backup antibiotics prescription (Martin *et al*., 2004); (c) enacting negative lists (eg, no intravenous transfusion for RTIs at primary care settings). In addition, the study identifies that age, type of residence, employment, medical insurance, annual food expenditure, and distance to medical facilities are factors affecting experience of and responses toward RTIs. These should also inform future countermeasures addressing the inappropriate use of healthcare. For example, the L-shaped link between service utilization rate and age suggests more attention on the increased use of healthcare by children but decreased use or delayed use by the elderly, while the non-linearity of delay in service use implies that a bell-shaped demand function should be used in health planning (Müller *et al*., 1998).

### Limitations of this study

This study has a number of limitations. First, there is a heavy reliance on self-report, which may be prone to bias due, for example, to issues of recall, particularly among the elderly; inability to distinguish RTI symptoms from other disease symptomatology; and possibly conforming with perceived expectations from the researchers (ie, the Hawthorn effect). The second limitation concerns seasonal differences in RTI prevalence. In general, RTI prevalence rates vary across seasons, with winter and spring being the time of year with the highest prevalence (Rafiefard *et al*., 2008; Aberle *et al*., 2010; Cicek *et al*., 2015). So readers are cautioned about potential seasonal bias.

## Conclusions

Despite the limitations, this study uncovered useful data for a better understanding RTI-related healthcare use among urban and rural residents in Anhui, China. Overall, >69% of informants who exhibited either the common cold, the nine AURTIs, and/or influenza sought clinical interventions. This can signify a marked issue with the large amount of healthcare for self-limited AURTIs.
